# Allergic Bronchopulmonary Aspergillosis in Children with Cystic Fibrosis: An Update on the Newest Diagnostic Tools and Therapeutic Approaches

**DOI:** 10.3390/pathogens9090716

**Published:** 2020-08-31

**Authors:** Claudia Lattanzi, Giulia Messina, Valentina Fainardi, Maria Candida Tripodi, Giovanna Pisi, Susanna Esposito

**Affiliations:** Pediatric Clinic, Pietro Barilla Children’s Hospital, Department of Medicine and Surgery, University of Parma, 43126 Parma, Italy; claudial1992@gmail.com (C.L.); messina.giulia@gmail.com (G.M.); valentina.fainardi@gmail.com (V.F.); mtripodi@ao.pr.it (M.C.T.); gpisi@ao.pr.it (G.P.)

**Keywords:** allergic bronchopulmonary aspergillosis, *Aspergillus fumigatus*, cystic fibrosis, lung disease

## Abstract

Cystic fibrosis (CF), the most common autosomal-recessive genetic disease in the Caucasian population, is characterized by frequent respiratory infections and progressive lung disease. Fungal species are commonly found in patients with CF, and among them, *Aspergillus fumigatus* is the most frequently isolated. While bacteria, particularly *Pseudomonas aeruginosa*, have a well-established negative effect on CF lung disease, the impact of fungal infections remains unclear. In patients with CF, inhalation of *Aspergillus* conidia can cause allergic bronchopulmonary aspergillosis (ABPA), a Th2-mediated lung disease that can contribute to disease progression. Clinical features, diagnostic criteria and treatment of ABPA are still a matter of debate. Given the consequences of a late ABPA diagnosis or the risk of ABPA overdiagnosis, it is imperative that the diagnostic criteria guidelines are reviewed and standardized. Along with traditional criteria, radiological features are emerging as tools for further classification as well as novel immunological tests. Corticosteroids, itraconazole and voriconazole continue to be the bedrock of ABPA therapy, but other molecules, such as posaconazole, vitamin D, recombinant INF-γ and Cystic Fibrosis Transmembrane Conductance Regulator (CFTR) modulators, have been showing positive results. However, few studies have been conducted recruiting CF patients, and more research is needed to improve the prevention and the classification of clinical manifestations as well as to personalize treatment. Early recognition and early treatment of fungal infections may be fundamental to prevent progression of CF disease. The aim of this narrative review is to give an update on ABPA in children with CF.

## 1. Background

*Aspergillus* species are ubiquitous saprophytic fungi found in water, soils, organic decay and in indoor environments. The commonest species associated to respiratory disease in humans is *Aspergillus fumigatus*. *Aspergillus* spores are inhaled daily, usually with no consequences, but, in susceptible people with pulmonary diseases such as cystic fibrosis (CF), the inhalation of *Aspergillus* conidia can colonize the lung and cause allergic bronchopulmonary aspergillosis (ABPA) [1]. ABPA is a Th2-mediated lung disease caused by hypersensitivity to *Aspergillus* hyphae that carries significant morbidity for patients with CF and may progress to bronchiectasis and pulmonary fibrosis. While bacterial infections, particularly those with *P*. *aeruginosa*, have a well-established negative effect on CF lung disease, the impact of fungal infections remains unclear [2], but an early diagnosis of ABPA seems clinically relevant in order to avoid deterioration of lung function [3].

The aim of this narrative review is to give an update on ABPA in children with CF, particularly regarding the newest diagnostic tools and therapeutic approaches.

## 2. Epidemiology of *Aspergillus fumigatus* in Patients with CF

Fungi are commonly found in patients with CF, and *Aspergillus fumigatus* is the most frequently isolated, with sputum samples found positive in 16% of children and adolescents [3] and up to 58% when also including older patients [4]. The use of specific fungal diagnostic protocols consisting of selective media for fungi and molecular methods has significantly increased *Aspergillus* detection in recent years [5]. In a recent study, Reece et al. [6] demonstrated that an extended incubation period and the use of quantitative polymerase chain reaction (qPCR) increased the detection of *A. fumigatus* in sputum samples from 16 to 52% of patients compared to the routine culture method.

Colonization of the airways can be suspected when *Aspergillus* is cultured in two or more samples over a year in patients without new respiratory symptoms and no specific fungal IgG in serum [5,7]. In younger children, data are unclear and colonization prevalence may be underestimated because this age group rarely produce a sputum and microbiological results rely on cough swabs. A retrospective study on 45 patients with CF analysed bronchoalveolar lavage (BAL) in very young children unable to expectorate and sputum in older children reporting an overall colonization prevalence of 22% with a higher risk over 8 years of age [8]. In an Australian cohort of preschool CF children, *Aspergillus* was the most frequently isolated pathogen in BAL on annual surveillance bronchoscopies, with a prevalence of 11% [9]. However, a positive culture for *Aspergillus* from sputum does not imply the development of ABPA because of the heterogeneity of the interaction between the fungus and the host. In fact, allergy to *Aspergillus* antigens detected by skin prick test is described in almost 50% of CF patients, whilst the prevalence of ABPA is estimated to be between 10% and 25% depending on the studies [10,11,12,13,14]. However, ABPA may be often underdiagnosed due to the lack of standardized diagnostic criteria.

## 3. Risk Factors for *Aspergillus* Colonization and Infection in Patients with CF

In CF, the main predisposing factor for colonization and eventually infection is impaired muco-ciliary clearance due to the dysfunction of Cystic Fibrosis Transmembrane Conductance Regulator (CFTR) that leads to a decrease in airway-surface liquid pH resulting in reduced antimicrobial activity and increased mucus viscosity [5].

Among colonized patients, many become sensitized to *A. fumigatus* with specific IgE or IgG, some produce a robust allergic response with high total IgE and high eosinophils [15] and only a small part develop ABPA [16]. The reasons why the clinical picture varies among patients are different and include clinical status, microbial interactions, medications, atopy and genetic immune host susceptibility.

A recent large multicentre study on 1541 subjects (mean age 15 years) with CF who developed chronic isolation of *Aspergillus* (two or more positive cultures during the 12-month period after the first positive result) demonstrated that pancreatic insufficiency was the greatest risk factor for *Aspergillus* infection, followed by use of macrolides and inhaled antibiotics and steroids [17]. In a study conducted in 26 children, low body mass index (BMI) led to a 10-fold increased risk of ABPA [18]. However, these findings are not conclusive since *Aspergillus* could be more frequent in patients with more severe disease and therefore requiring more long-term antibiotic therapies. It has been argued that aggressive antibiotic therapies such as long-term azithromycin and chronic inhaled antibiotics in early age may have decreased the prevalence of *S*. *aureus* and *Pseudomonas aeruginosa* but increased *Aspergillus* airway colonization, as shown by Breuer et al. [9]. The mechanism behind the association between long-term therapy with macrolides and risk of *Aspergillus* colonization would be the inhibitory effect of azithromycin on the recruitment and activation of neutrophils, the first-line defences against *Aspergillus* [18]. With regard to genetic predisposing factors, HLA DR2 and DR5, and possibly DR4 and DR7, have been suggested to increase the risk for ABPA, whereas HLA DQ2 seems to be protective [19,20]. Furthermore, Brouad et al. reported a specific genotype in the IL-10 promotor region as a predisposing factor of *Aspergillus* colonization and ABPA development [21]. Many associations between ABPA and SNPs for IL-4 receptor, IL-13 and Toll-like receptor (TLR) 3 have been found in people without CF suggesting that genetic predisposition can be the cause of the aberrant response to *A. fumigatus* and susceptibility to ABPA seen in certain individuals [22].

Moreover, alterations in the airway microbiome may be a predisposing factor for fungal colonization and development of ABPA in patients susceptible to aspergillosis [23] and these alterations might be enhanced by the frequent use of antibiotics in patients with CF [24]. In summary, the interplay between *Aspergillus* virulence and host-susceptibility determines the onset and the course of the disease.

## 4. Pathogenesis of *Aspergillus fumigatus* Infection in Patients with CF

It is well established that ABPA is caused by hypersensitivity to *Aspergillus* antigens but why only some patients develop the allergy and the disease remains unclear

In susceptible hosts, inhaled spores of *Aspergillus* germinate to hyphae and persist into the lungs, leading to the damage of muco-ciliary clearance and epithelium barrier and eventually activating a strong type 2 immune response [25,26]. Type 2 immune response is characterized by the production of IL-4, IL-5 and IL-13 from innate lymphoid cells, Th2 cells and eosinophils, mast cell degranulation, eosinophil activation with an increase in total IgE, *Aspergillus*-specific IgE and the production of *Aspergillus*-specific IgG antibodies [10,18]. The consequent pulmonary eosinophilic inflammation results in recurrent pulmonary infiltrates and eventually in bronchiectasis and pulmonary fibrosis.

In patients with altered lung structure, such as CF patients where the innate defense of mucociliary clearance is impaired, *Aspergillus* spores penetrate and adhere to collagen and fibronectin fibres in the basal lamina, facilitating its persistence into the airways [27]. Moreover, the fungal metabolite gliotoxin has been shown to downregulate vitamin D receptor expression in macrophages and airway epithelial cells of CF patients and increase the levels of IL-5 and IL-13 [28]. In CFTR-deficient mice, even in the absence of ABPA, *Aspergillus* elicits an aberrant Th2 immune response with increased levels of IL-4, IL-13 and IgE [28].

Allergenic proteinases secreted by *Aspergillus* are considered the main pathogenic factor that facilitates the fungal persistence in respiratory airways. *Aspergillus* proteinases are recognized by the innate immune receptors TLR 2, TLR4 and TLR6 resulting in the initiation of allergic airway inflammation with activation of both innate and adaptive immunity and release of pro-inflammatory cytokines [29,30,31].

Although eosinophils have been traditionally implicated in fighting fungal infections, the latest evidence attributes to Th2 cytokines an important role in this hypersensitivity reaction. Flow cytometer studies on peripheral blood of CF patients with ABPA showed a skewing towards Th2 cells with a reduction in IFNg production and murine models confirmed that CFTR mutation is associated with an increased sensitivity to IL-4 [28,32].

Dietschmann et al. concluded that T-cell-derived IL-4/IL-13 is essential for *A*. *fumigatus*-induced lung eosinophilia and inflammation, while eosinophils seem to have immunomodulatory and not only inflammatory function [33]. Taken together, these results suggest that the increased susceptibility of CF patients to ABPA might be due to an exaggerate Th2 response and a deficient Th1 response.

Nevertheless, a clear understanding of the pathophysiological mechanisms determining allergic and chronic forms of pulmonary aspergillosis such as ABPA has been limited by the lack of reliable murin models of *Aspergillus* colonization, the prerequisite of fungal infection [34].

## 5. Clinical Features of ABPA in Patients with CF

In CF, the interaction between the host and *A*. *fumigatus* can range from asymptomatic conditions to rapidly invasive disseminated diseases, as follows: (1) colonization, (2) serological sensitization (3) *Aspergillus* bronchitis, (4) chronic pulmonary aspergillosis or (5) allergic aspergillosis. These diseases can present as ABPA, *Aspergillus*-induced asthma (AIA) or allergic *Aspergillus* sinusitis (AAS). One form of clinical disease may evolve into another. Colonization is defined when two or more respiratory samples are positive for *Aspergillus* by culture or PCR in patients without new respiratory symptoms and no fungal specific IgG in serum [7]. Fungal bronchial colonization leads to airway inflammation and eventually to the development of allergic forms of aspergillosis [35]. The presence of serum IgE specific to *Aspergillus* without symptoms describes the fungal serological sensitization; this can be associated with a skin prick test positive for *Aspergillus*. *Aspergillus* bronchitis is a chronic infection of the lower respiratory airways that may affect about 9% of CF individuals [36]. Symptomatic patients with a positive culture for *Aspergillus* (or PCR), *Aspergillus*-specific IgG or precipitins but that do not fulfil the diagnostic criteria for ABPA or invasive aspergillosis may have *Aspergillus* bronchitis. Chronic pulmonary aspergillosis is characterized by progressive cavitation, fibrosis and pleural thickening; a fungal ball called aspergilloma may appear in the cavity [25]. There is a strong association between nontuberculous mycobacterial infection and chronic pulmonary aspergillosis [35]. ABPA can present with great variability; patients can initially be asymptomatic or slightly symptomatic with fatigue, persistent mild cough, increased sputum, haemoptysis, fever, malaise and exercise-induced dyspnoea or, conversely, the onset might be acute and seldom fulminant [26,35]. The progression of ABPA can be summarized in five stages, as reported in Table 1 [37].

The mechanisms determining the predisposition towards certain clinical manifestations are not yet fully understood, but the pathogenicity of the fungus and the host immunity are supposed to be the main implicated factors.

## 6. ABPA and Lung Function

The impact of ABPA on the decline in lung function remains unclear. In their longitudinal data analysis of 3350 patients, Kaditis et al. found that the difference in forced expiratory volume in one second (FEV1) between children with and without ABPA was modest. During a 3-year follow-up, FEV1 was significantly lower in children with ABPA during the first year; no significant difference was observed in the subsequent years [38]. De Baets et al. performed a retrospective case-control study in 73 *P*. *aeruginosa-*negative patients and found that patients with ABPA had significantly lower FEV1 values. They suggested that this decline in lung function preceded ABPA diagnosis by approximately 2 years [39]. When other factors were analysed, a low BMI Z-score was found to have the greatest impact on the progression of lung disease followed by high-risk genotype, female sex, CF-related diabetes mellitus, chronic *P*. *aeruginosa* infection and, last, ABPA [38].

A cross-sectional analysis carried out by Harun et al. in 5- to 14-year-old children with BAL positive for *Aspergillus fumigatus* reported increased chest CT scores for air trapping at age 5 years [40]. Similarly, an interesting study by Breuer et al. in 330 children with CF demonstrated that *Aspergillus* infection was associated to progression of structural lung disease (especially air trapping) at annual chest CT scan and to increased risk of hospitalization [41]. Both studies reported no association between the presence of *Aspergillus* in BAL and lung function.

At present, there is no consensus for the treatment of asymptomatic *Aspergillus* colonization in CF, but numerous studies have concluded that patients chronically colonized with *A. fumigatus* have a more rapid decline in FEV1 than non-colonized patients [3,8,42]. Furthermore, co-colonization between *A. fumigatus* and *P. aeruginosa* is associated with poorer clinical outcomes [43].

## 7. Diagnosis of ABPA in Patients with CF

Diagnosis of ABPA in CF patients remains challenging because ABPA can overlap other syndromes, such as chronic pulmonary aspergillosis or, when complicated by bronchiectasis, *Aspergillus* bronchitis [13]. In the non-CF population, the presence of bronchiectasis and chest infiltrates can orientate the clinician for a diagnosis of ABPA, but, in CF, these features are typical, making the distinction between type 2 high fungal allergen-exacerbated asthma very difficult. Moreover, there is no consensus regarding the cut-off values for the diagnostic serological tests in CF because most studies have been performed in asthma patients [35]. Over the last 50 years, several diagnostic criteria have been proposed, but a consensus has not been reached [37]. To date, four different working groups have proposed diagnostic criteria in order to help clinicians in identifying this entity.

The Rosenberg and Patterson criteria included eight major (asthma, transient pulmonary infiltrates, immediate cutaneous reactivity to *A*. *fumigatus*, elevated total serum IgE, precipitating antibodies against *A*. *fumigatus*, peripheral blood eosinophilia, elevated serum IgE and IgG to *A*. *fumigatus*, central/proximal bronchiectasis with normal tapering of distal bronchi) and three minor criteria (expectoration of golden-brownish sputum plugs, positive sputum culture for *Aspergillus* species, late skin reactivity to *A*. *fumigatus*), as shown in Table 2. The presence of six out of eight major criteria makes diagnosis almost certain.

In 2003, the Cystic Fibrosis Foundation held a consensus conference to revise pre-existing criteria. They identified five criteria as follows: acute or subacute clinical deterioration in pulmonary function, serum total IgE concentration >1000 IU/mL unless the patient is receiving systemic corticosteroids, positive serum-specific IgE (>0.35 kUA/L) or immediate skin test, precipitating antibodies to *A*. *fumigatus* or serum IgG antibodies to *A*. *fumigatus* by an in vitro test, and new or recent infiltrates (or mucus plugging) on radiological imaging that do not respond to antibiotics and standard physiotherapy. The criteria are shown in Table 3 [14].

In 2013, the ISHAM Working Group defined predisposing conditions such as asthma or CF and then established obligatory criteria consisting of: (1) immediate cutaneous reactivity to *A. fumigatus* or elevated IgE levels against *A. fumigatus* and (2) elevated total IgE levels >1000 IU/mL. They are both needed for diagnosis. In addition, they proposed the following other criteria: (1) the presence of IgG antibodies against *A. fumigatus* or precipitating antibodies, (2) the presence of pulmonary opacities on chest X-ray and/or (3) eosinophil count >500/µL in steroid-naïve patients [16]. Two out of the three criteria are needed for diagnosis. Obligatory criteria and other criteria are presented in Table 4.

Maleki et al. found no significant effect on the reported prevalence of ABPA in CF patients depending on the applied criteria [45].

Recently, Denning DW et al. suggested a simplified scheme to recognize fungal colonization and sensitisation from ABPA (Table 5, adapted from Denning et al. [46]).

The same group proposed a new classification for aspergillosis in CF patients that integrates in the diagnostic criteria sputum galactomannan and real-time *Aspergillus* PCR with *Aspergillus* serum-specific IgE and IgG. This classification intends to recognize *Aspergillus* bronchitis from simple colonization and to differentiate *Aspergillus* sensitization from ABPA [47]. Latent class analysis of 146 adult patients with CF identified three distinct classes of aspergillosis: ABPA, patients with a positive PCR, elevated total and specific *A. fumigatus* IgE/IgG and a positive galactomannan; *Aspergillus* sensitized, patients with or without a positive PCR, elevated *A. fumigatus* IgE (not IgG) and a negative sputum galactomannan; *Aspergillus* bronchitis, patients with a positive PCR, elevated A. fumigatus IgG and a positive sputum galactomannan. Interestingly, 17% of patients moved between classes over a 9-month period of follow-up highlighting the known variability of *Aspergillus* disease [47].

Together with clinical manifestations and laboratory findings, radiological features can help with the diagnosis. Moreover, chest X-rays and CT scan findings allow further classification of ABPA as follows: (1) ABPA-seropositive (ABPA-S) when patients meet the minimum criteria but without central or peripheral bronchiectasis; (2) ABPA-central bronchiectasis (ABPA-CB) when patients meet the minimum criteria with central bronchiectasis; (3) severe asthma associated with fungal sensitivity (SAFS) when patients with asthma and sensitivity to fungi do not meet the minimum criteria for ABPA [48,49]. A recent study by Lu et al. analysed 125 ABPA-CB patients and found that clinical course and prognosis correlated with the radiological phenotype of mucus plugs [50]. Based on their density on CT, mucus plugs have been classified in high-attenuation mucus (HAM) and low-attenuation mucus (LAM). The authors have found that the presence of HAM positively correlates with the severity of the disease. In addition to the lack of unique criteria, ABPA in CF patients can be associated with other respiratory infections caused by both fungi and bacteria, which can themselves determine a decline in impaired lung function, making diagnosis even harder. Many examples of delayed diagnosis due to co-infections can be found in the literature. A recent case report by an Italian group described a case of delayed diagnosis in a 10-year-old child co-infected with *Aspergillus* and *Mycoplasma pneumoniae* [51]. Since clinical manifestations might be misleading, there is great need for novel diagnostic tools. Recent studies have highlighted that the use of basophil activation test (BAT) and lymphocyte stimulation test (LST) could both enhance the diagnosis of ABPA [52]. BAT explores immediate hypersensitivity mechanisms by evaluating basophil activation ex vivo, whilst LST investigates delayed hypersensitivity responses. In particular, the level of basophil activation has shown correlation with the impairment of lung function and therefore could possibly be used with a prognostic value [53]. Likewise, the grade of CD4+ and CD8+ activation seems to be fairly accurate in predicting the development of ABPA. These findings are still under investigation as further studies are needed. Another functional test found to be useful in diagnosing and monitoring the course of ABPA before and after treatment is thymus and activation-regulated chemokine (TARC), whose serum levels are higher in ABPA patients and decrease after therapy [53].

Large longitudinal studies are required to define worldwide standardized clinical and immunological criteria to diagnose ABPA and to assess the relationship between these criteria and effective lung damage [13].

## 8. Treatment of ABPA in Patients with CF

The aim of ABPA treatment is to control symptoms, prevent and treat exacerbation, limit lung inflammation and reduce progression to end-stage pulmonary disease. The consequences of delayed treatment are fibrosis, bronchiectasis and loss of lung function [54].

Few studies have been conducted recruiting CF patients; therefore, clinical practice relies on the experience acquired in asthma.

The core of the therapy relies on corticosteroids to control the inflammatory process and azoles to reduce the fungal burden. In Table 6, we report the proposed therapeutic scheme of oral corticosteroids in CF patients with ABPA.

A reduction in serum IgE levels of 25% after one month of therapy and 60% after two months of therapy is expected. A serum IgE level decrease of at least 35% is considered a good therapeutic response [56].

In the last few decades, there has been increasing evidence that antifungal therapy with azoles may play a significant role in the management of ABPA in patients with CF [57], reducing the antigenic stimulus of the fungus and therefore the inflammation cascade [58]. Currently, the most studied molecules are itraconazole and voriconazole. Itraconazole increases corticosteroid plasma levels through a reduction in corticosteroid metabolism, thus allowing for a reduction in oral corticosteroid dosage but also exposing patients under inhaled corticosteroids to unexpected systemic concentrations [59,60]. Itraconazole bioavailability varies among individuals and monitoring of serum concentrations is recommended to maintain the right exposure. Voriconazole has better oral bioavailability and is better tolerated (fewer gastrointestinal adverse effects), but phototoxicity has been reported in up to 20% of children, and this percentage doubles if treated for 6 months or longer [61]. Of note, an association with skin cancer has been described with its long-term use [62,63]. Due to the increased use of triazole drugs, resistant strains of *A. fumigatus* have been selected [64]. There is emerging evidence that posaconazole can be a valid alternative therapy. The resistance profile is similar to itraconazole, but absorption and tolerance are greater. A retrospective study in 32 patients with CF suggested that posaconazole is more effective than itraconazole and voriconazole at reducing *A. fumigatus* IgE in ABPA [65]. In a prospective study of 14 children with CF, posaconazole treatment was well tolerated and resulted in a modest but significant improvement in lung function [66]. A case report described a patient with ABPA successfully treated with a new triazole called isavuconazole without serious adverse effects [67]. Randomized controlled studies are needed to evaluate which is the best antifungal treatment for ABPA in patients with CF. Nebulized amphotericin B has been used as a treatment for invasive *Aspergillus* infection. Therefore, its use has been proposed to treat difficult cases of ABPA. As bronchospasm and cough have been reported as side effects, this medication should be administered under medical surveillance [63].

Since IgE-mediated hypersensitivity is involved in ABPA pathogenesis, omalizumab (i.e., a humanized monoclonal antibody against IgE) use has been proposed for ABPA in CF patients [68,69]. Perisson et al. retrospectively analysed 18 patients and observed a stabilization in lung function decline after the initiation of biological therapy with omalizumab along with a notable reduction in the corticosteroid daily dose needed and an improvement in the nutritional status [70]. Similarly, the use of mepolizumab, a humanized monoclonal antibody for IL-5, has been reported in three patients with CF and ABPA and demonstrated good tolerability and a beneficial effect in reducing oral corticosteroids [71]. However, there is a lack of strong evidence for the use of humanized monoclonal antibodies in people with CF and ABPA, and further studies are needed.

CF patients with ABPA were found to have lower levels of serum vitamin D. The therapeutic role of vitamin D in ABPA has been investigated. The addition in vitro of 1,25 OH-vitamin D3 reduced Th2 response and the expression of OX40 ligand on dendritic cells, responsible for the Th2 response mediated by innate airway epithelium cytokines, and potentiated Treg-mediated regulation of Th2 reactivity [72]. A phase I clinical trial demonstrated the efficacy of a daily supplementation with 4.000 UI of vitamin D for 24 weeks in reducing IL-13-mediated Th2 response and *Aspergillus*-specific IgE levels in CF children aged 12 years with ABPA [73].

IFN-γ increases the killing capacities of human neutrophils and monocytes against *A. fumigatus* hyphae and *A. terreus* and the release of pro-inflammatory cytokines. Recombinant IFN-γ therapy was therefore used in several clinical trials to treat or at least to prevent fungal infections.

In recent years, promising data came from CFTR modulator drugs that significantly reduced *Aspergillus* in respiratory cultures [74].

More research is required to determine which is the right treatment and whether anti-fungal therapy influences the course of disease.

## 9. Conclusions

CF patients with ABPA should undergo regular follow-up. The serum IgE level is the most commonly used parameter to assess the benefit of the treatment. A serum IgE level decrease of at least 35% is considered a good therapeutic response; doubled IgE levels at any stage indicate an ABPA exacerbation [56]. Given the consequences of a late ABPA diagnosis, it is imperative that the diagnostic criteria guidelines be reviewed and standardized. Moreover, the availability of certain diagnostic criteria is essential to limit the risk of ABPA overdiagnosis. Early treatment of *Aspergillus* colonization could prevent the progression to ABPA and limit pulmonary damage. However, colonized patients that may not develop ABPA in the future might be exposed to unneeded corticosteroid therapy. Future research studies are needed to prevent *Aspergillus* colonization, improve the classification and diagnosis of clinical manifestations and find personalized treatments.

In conclusion, the real impact of ABPA in CF patients requires further investigations. Studies on paediatric population are limited and clinical practice is mostly based on experience with adults. Since ABPA is a rare entity in children, we suggest that multicentre studies are implemented in order to have larger cohorts of patients and therefore more conclusive evidence. Moreover, laboratory testing should be standardized to objectively compare results among centres.

## Figures and Tables

**Table 1:** Stages of allergic bronchopulmonary aspergillosis (ABPA), adapted from Patel et al. [37].

Stage
**Stage I, acute** The patient is diagnosed with all typical features (*Aspergillus*-specific IgE, radiological abnormalities, peripheral blood eosinophilia, *Aspergillus*-specific serum precipitins)
**Stage II, remission** Asymptomatic patient, no new radiological infiltrates, no rise in total IgE for at least 6 months
**Stage III, exacerbation** New pulmonary infiltrates on chest X-ray with peripheral blood eosinophilia and double the remission IgE level
**Stage IV, corticosteroid-dependent asthma** Inability to taper off from corticosteroid treatment
**Stage V, fibrotic lung disease** Irreversible fibrosis and chronic cavitation at X-ray and CT scan

**Table 2:** Diagnostic criteria for allergic bronchopulmonary aspergillosis (ABPA) according to Rosenberg-Patterson, adapted from Shah A et al. [44].

Diagnostic Criteria
**Major** Asthma Presence of transient pulmonary infiltrates (fleeting shadows Immediate cutaneous reactivity to *A*. *fumigatus* Elevated total serum IgE Precipitating antibodies against *A*. *fumigatus* Peripheral blood eosinophilia Elevated serum IgE and IgG to *A*. *fumigatus* Central/proximal bronchiectasis with normal tapering of distal bronchi
**Minor** Expectoration of golden-brownish sputum plugs Positive sputum culture for *Aspergillus* species Late (Arthus-type) skin reactivity to *A*. *fumigatus*

**Table 3:** Diagnostic criteria for allergic bronchopulmonary aspergillosis (ABPA) according to the Cystic Fibrosis Foundation Consensus Conference, adapted from Stevens DA et al. [14].

Diagnostic Criteria
Cystic fibrosis with acute or subacute clinical deterioration Serum total IgE concentration >1000 IU/mL unless the patient is receiving systemic corticosteroids Positive serum-specific IgE (>0.35 kUA/L) or immediate skin test Precipitating antibodies to *A*. *fumigatus* or serum IgG antibodies to *A*. *fumigatus* by an in vitro test New or recent infiltrates (or mucus plugging) on chest radiography or CT that do not respond to antibiotics and standard physiotherapy

**Table 4:** Diagnostic criteria for allergic bronchopulmonary aspergillosis (ABPA) according to the ISHAM Working Group, adapted from Agarwal R et al. [16].

Diagnostic Criteria
**Obligatory (both needed)** Type 1 *Aspergillus* skin test positive or elevated IgE against *A. fumigatus* Elevated total IgE levels (>1000 IU/mL unless all other criteria are met)
**Other (≥2 out of 3):** Presence of IgG antibodies against *A. fumigatus* or precipitating antibodies Presence of fleeting or fixed pulmonary opacities on chest X-ray Eosinophil count >500 cells/µL in steroid-naïve patients

**Table 5:** Definitions for fungal colonization, fungal sensitisation and allergic bronchopulmonary aspergillosis (ABPA) according to Denning DW et al [46].

	Proposed Definition
**Fungal colonization**	One (preferably two or more) respiratory samples positive for *Aspergillus* by colture or PCR No new major respiratory symptoms No evidence of ABPA or other forms of Aspergillosis No overt immunocompromised Negative serum *Aspergillus* IgG
**Fungal sensitization**	*Aspergillus* skin test positive or elevated IgE against *A. fumigatus* without inflammation or lung damage
**ABPA**	*CF* *Aspergillus* skin test positive or elevated IgE against *A. fumigatus* Elevated total IgE levels (>1000 IU/mL)

**Table 6:** Proposed corticosteroid regimen for allergic bronchopulmonary aspergillosis (ABPA), adapted from Ohn at al [55].

Regimen
Weeks 0–2 Prednisolone 1 mg/kg/day (max 50 mg/die)
Weeks 2–4 Prednisolone 0.5 mg/kg/day
Weeks 4–6 Prednisolone 0.5 mg/kg 3 times per week
Weeks 6–8 Prednisolone 0.5 mg/kg 3 times per week
